# Predicting the potential distribution of Astragali Radix in China under climate change adopting the MaxEnt model

**DOI:** 10.3389/fpls.2024.1505985

**Published:** 2024-12-06

**Authors:** Zixuan Wen, Ke Yan, Man Zhang, Ruiqing Ma, Xiaoyan Zhu, Qing Duan, Xiaolin Jiang

**Affiliations:** ^1^ School of Public Health, Shandong Second Medical University, Weifang, China; ^2^ Department of Science and Education, Shandong Center for Disease Control and Prevention, Jinan, China; ^3^ AIDS Prevention and Control Section, Shandong Center for Disease Control and Prevention, Jinan, China; ^4^ Infectious Disease Prevention and Control Section, Shandong Center for Disease Control and Prevention, Jinan, China

**Keywords:** Astragali Radix, maximum entropy model, geographic information system, suitable area, environmental factor, climate change

## Abstract

**Introduction:**

Astragali Radix is the dried root of Astragalus mongoliae or Astragalus membranaceus, a leguminous plant. Since ancient times, Astragali Radix has been widely used in Chinese traditional Chinese medicine. As people become more health-conscious, the market demand for Astragali Radix grows and its popularity is increasing in the international market. As an important medicinal plant, the growth of Astragali Radix is strongly influenced by environmental conditions. In order to meet the market demand for high quality Astragali Radix herbs, it is necessary to search and find areas suitable for the growth of Astragali Radix.

**Methods:**

In this study, we assessed the potential impacts of climate change on the distribution of the Chinese medicinal plant Astragali Radix using the maximum entropy (MaxEnt) model in combination with a geographic information system(GIS). Distribution data and environmental variables were analyzed to predict suitable areas for Astragali Radix under the SSP126, SSP245 and SSP585 scenario for current and future (2041-2060, 2061-2080, 2081-2100). Jackknife is used to assess the importance of environmental variables, and environmental variables with a model contribution greater than 5% were considered to be the main drivers.

**Results:**

The results showed that the current area of suitable area for Astragali Radix is 188.41 km^2^, and the three climate scenarios show an increasing trend in the future, with a maximum of 212.70 km^2^. North China has always been the main suitable area, while the area of suitable area in Southwest China is decreasing, and Xinjiang will be developed as a new suitable area in the future. Annual precipitation (41.6%), elevation (15.9%), topsoil calcium carbonate (14.8%), annual mean temperature (8.3%), precipitation seasonality (8%) and topsoil pH (6%) contributed more to the model and were the main environmental influences on the distribution of Astragali Radix. In addition, the centroids of the suitable areas shifted northward under all three climate scenarios, indicating a migratory response to global warming.

**Discussion:**

Our study found that suitable area of Astragali Radix has been expanding for most of the time in each period of the three climate scenarios compared with the current situation. In the future, humans can focus on enhancing the cultivation techniques of Astragali Radix in these suitable areas. This study provide a scientific basis for the development of planting strategies and spatial distribution management of Astragali Radix. It helps to optimize the selection of planting areas and resource conservation of Chinese herbs.

## 1 Introduction

Astragali Radix is the dried root of Astragalus mongoliae or Astragalus membranaceus, a leguminous plant, which not only has good medicinal value of tonic qi, but also has the ecological functions of windbreak, sand fixation and soil and water conservation (Zhong, 2018). It is common in Eurasian countries, including China, Russia, Kazakhstan and Mongolia (Zhang, 2007). In China, Astragali Radix is mainly distributed in Heilongjiang, Jilin, Nei Mongol, Hebei, Ningxia, Gansu, Qinghai, Sichuan, and Tibet (Liu et al., 2019; Wang et al., 2023c). The polysaccharides, saponins, and flavonoids of Astragali Radix are its main active components and the main basis for evaluating the quality of Astragali Radix herbs (Zhang et al., 2022). which make Astragali Radix have a variety of medicinal values such as improving immunity, protecting the liver, nourishing and tonifying (Ma et al., 2022; Shao et al., 2023), and have been widely used in the treatment of hypoxic-ischemic encephalopathies, circulatory disorders, respiratory disorders, renal disorders, neurological disorders, and blood sugar and blood pressure. China has focused on ecological protection in recent years and advocated standardized cultivation of Chinese herbs, which has provided an opportunity for the scientific cultivation of Astragali Radix and many other Chinese medicinal plants (National Forestry and Grassland Administration, 2022).

Climatic, meteorological, topographical and geomorphological factors are of vital importance to plant cultivation, and they not only influence the growth and development of plants, but also determine their distribution range and planting strategies. The cultivation and efficacy of herbal medicines, in particular, are greatly influenced by ecological factors (Wu et al., 2019). Soil is a factor that must be considered when conducting research on plant cultivation. Previous scholars have conducted several studies on the relationship between the active ingredients of medicinal plants and soil factors (Shang et al., 2012; Hou et al., 2024; Yang et al., 2024). These studies all indicate that soil factors affect the accumulation of active ingredients in drugs. Furthermore, the geographic distribution pattern of plants will change as global temperatures rise, precipitation patterns change, and extreme weather events become more frequent (Kosanic et al., 2018). Climatic spaces suitable for plant distribution are likely to disappear (Gómez-Ruiz and Lacher, 2019), accompanied by changes in the topography suitable for medicinal plants (Li et al., 2024a). A study on the geographic distribution of plant species in China shows that 122 plant species in China are at risk of losing their geographic ranges completely under future climate change scenarios, and 125 species will migrate to higher latitudes and higher altitudes (Wang and Wu, 2024). The same migration trend was confirmed in a study by Malaysian scholars (Muzafar et al., 2022).

Ecological niche models (ENMs) are an important tool for speculating the potential distribution areas of species by comprehensively analyzing their distribution information and related environmental variables (Zhang et al., 2024b), and a variety of models have been derived, such as the Maximum Entropy Model (MaxEnt) (Zhao et al., 2022b), the biological population growth model (CLIMEX) (Early et al., 2022), the Ecological Niche Factor Analysis (ENFA) (Rosas et al., 2022), and the Genetic Algorithm Model (GAM) (Liu et al., 2012). Among them, the MaxEnt model proposed by Edwin Thompson Jaynes in 1957 is considered to be the best tool to be used in conjunction with GIS (Geographic Information System). It is a probabilistic model based on entropy maximization, which can learn conditional probability distributions by maximizing entropy under given conditions, and is able to predict the potential range of a species using known species distribution data and environmental factors (Wang et al., 2024). Due to its simplicity in modeling and accuracy in prediction, the MaxEnt model has been widely used in the fields of ecology, geography and botany in recent years. In particular, it plays an important role in the prediction of potentially suitable areas of species (Xia et al., 2023; Chen et al., 2022), the risk assessment of biological invasion (Wang et al., 2023a), and the prediction of pest and disease trends (Chen et al., 2024). Today, the MaxEnt model has been used in the distribution studies of a variety of herbal medicines, such as gastrodia elata (Hu et al., 2023), trollius wildflowers (Fan and Luo, 2024), gymnadenia conopsea (Cha et al., 2024), cinnamomum cassia (Li et al., 2024b), and so on. This provides a scientific basis for predicting the impact of climate change on the distribution of Chinese herbal medicines in suitable areas, evaluating the amount of Chinese herbal medicine resources, and guiding the selection of areas for cultivation of Chinese herbal medicines.

In recent years, wild Astragali Radix resources have been decreasing year by year, and nowadays artificial cultivation is the mainstay (Zhang et al., 2024a). Therefore, in order to conserve and cultivate Astragali Radix, it is crucial to understand its current regional distribution in China and its spatial pattern of suitable areas under future climate change. In this study, we combined the MaxEnt model and Geographic Information System (GIS) to screen the dominant environmental factors affecting the potential distribution of Astragali Radix on the basis of the available distribution information, taking into account climate, topography and soil factors (Xu et al., 2023). Meanwhile, the changes in the distribution of the possible suitable areas and the migration of centroids of Astragali Radix were predicted under the scenarios of SSP126, SSP245, and SSP585 for the years 2041-2060, 2061-2080, and 2081-2100, respectively. This study provides an important reference for future conservation and cultivation strategies of Astragali Radix.

## 2 Materials and methods

### 2.1 Data collection

Astragali Radix distribution data were obtained from the Global Biodiversity Information Facility (GBIF, http://www.gbif.org/) and the Chinese Virtual Herbarium (CVH, http://www.cvh.ac.cn/). Bioclimatic variables were downloaded from the World Climate Database (WorldClim, http://www.worldclim.Org/). Elevation data were obtained from the Geospatial Data Cloud (http://www.gscloud.cn/) of the Chinese Academy of Sciences, from which slope and aspect were extracted. Soil data were obtained from the Harmonized World Soil Database (Harmonized World Soil Database version 1.1, http://www.fao.org/soilsportal/). A total of 35 environmental variables were used in our study, including 19 climate factors, 13 soil factors, and 3 topographic factors, and all data had a spatial resolution of 2.5min (**Table 1**). Where bioclimatic variables included current data and future data (2041-2060, 2061-2080, 2081-2100) under the Shared Socioeconomic Pathway 1-2.6 (SSP126), the Shared Socioeconomic Pathway 2-4.5 (SSP245) and the Shared Socioeconomic Pathway 5-8.5 (SSP585). SSP126, SSP245, and SSP585 represent future climate scenarios with radiative forcing targets of 2.6, 4.5, and 8.5 W/m², respectively, and they represent different global development paths from active climate policies to high-emission trajectories.

**Table 1:** The contribution rate of environmental variables.

Category	Abbreviation	Variable	Whether excluding	Percent contribution (%)	Suitable range
Climate	bio1	Annual mean temperature (°C)	No	8.3	4.86~15.08
	bio2	Mean diurnal range (°C)	No	1.6	9.41~13.50
	bio3	Isothermality (bio2/bio7) (×100)	No	1.5	24.30~32.26
	bio4	Temperature seasonality	Yes	–	–
	bio5	Max temperature of warmest month (°C)	Yes	–	–
	bio6	Min temperature of coldest month (°C)	Yes	–	–
	bio7	Temperature annual range (°C)	Yes	–	–
	bio8	Mean temperature of wettest quarter (°C)	No	0.1	18.29~25.22
	bio9	Mean temperature of driest quarter(°C)	Yes	–	–
	bio10	Mean temperature of warmest quarter (°C)	Yes	–	–
	bio11	Mean temperature of coldest quarter (°C)	Yes	–	–
	bio12	Annual precipitation (mm)	No	41.6	351.96~712.77
	bio13	Precipitation of wettest month (mm)	Yes	–	–
	bio14	Precipitation of driest month (mm)	Yes	–	–
	bio15	Precipitation seasonality	No	8.0	-165.68~100.23
	bio16	Precipitation of wettest quarter (mm)	Yes	–	–
	bio17	Precipitation of driest quarter (mm)	Yes	–	–
	bio18	Precipitation of warmest quarter (mm)	Yes	–	–
	bio19	Precipitation of coldest quarter (mm)	Yes	–	–
Soil	t_bs	Topsoil base saturation (%)	Yes	–	–
	t_caco3	Topsoil calcium carbonate (% wt.)	No	14.8	2.08~16.38
	t_caso4	Topsoil Gypsum (% wt.)	No	0	-1.51~0.34
	t_clay	Topsoil clay fraction (% wt.)	No	0.2	8.00~24.67
	t_ece	Topsoil salinity (dS/m)	No	0	0.14~1.63
	t_esp	Topsoil sodicity (%)	No	0.3	0.49~4.72
	t_gra	Topsoil gravel content (%vol.)	No	0	2.99~9.72
	t_oc	Topsoil organic carbon (% wt.)	No	0	-3.75~-0.91
	t_ph	Topsoil pH (-log (H+))	No	6.0	7.31~9.34
	t_ref	Topsoil reference bulk density (kg/dm3)	Yes	–	–
	t_sand	Topsoil sand fraction (% wt.)	No	0.2	28.99~50.64
	t_silt	Topsoil silt fraction (% wt.)	Yes	–	–
	t_teb	Topsoil TEB (cmol/kg)	No	0.1	16.79~65.30
Topographical	d_ele	Elevation (m)	No	15.9	-783.10~1052.71
	d_asp	Aspect (rad)	No	0.4	154.75~352.65
	d_slo	Slope (°)	No	1.0	-3.24~2.05

### 2.2 Data processing

#### 2.2.1 Analysis and processing of occurrence data

To avoid errors caused by clustering effects, the 802 Astragali Radix distribution data were filtered using the ENMTools 1.4 tool so that only one observation was retained in each 2.5 min grid. After removing redundant data, the final 195 distribution data were included in the model.

#### 2.2.2 Analysis and processing of environmental variables

**Figure 1:**
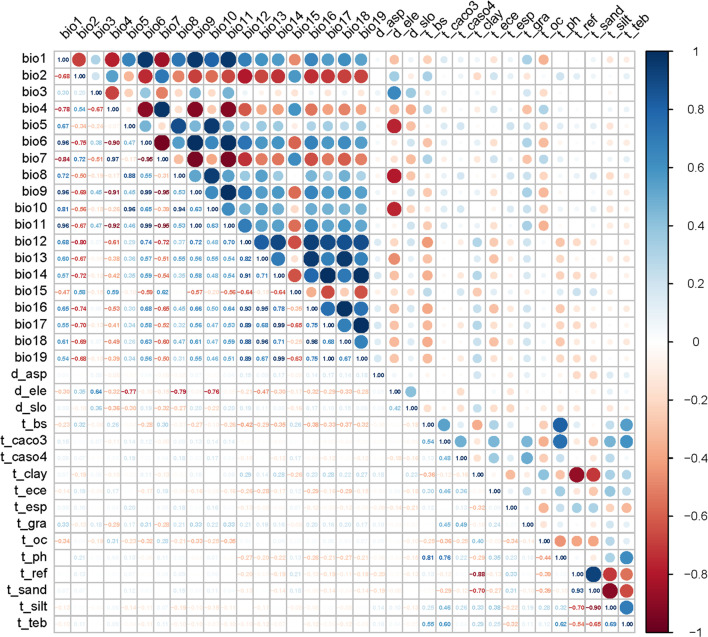
Correlation of environment variables.

The 35 environmental data corresponding to the location of Astragali Radix were extracted using ArcGIS 10.7 software, and Pearson correlation analysis was performed using SPSS 25.0 software. For the environmental variables with correlation coefficients greater than 0.8 in the results, only one was retained (the one with higher contribution was selected), and finally 19 environmental variables were included in the model (**Figure 1**; **Table 1**).

### 2.3 Model evaluation

The ENMeval package in R 4.3.1 was used for parameter optimization to improve the accuracy of MaxEnt model by adjusting the optimal values of the regularization multiplier (RM) and the feature categories (FC) (Muscarella et al., 2014). FC (H, L, LQ, LQH, LQHP, LQHPT) and RM (0.5, 1, 1.5, 2, 2.5, 3, 3.5, 4) were combined to calculate the Akaike Information Criterion Coefficients (AICc) and to assess the model fit and complexity. The optimal combination of parameters with the minimum AICc score (DAICc=0) was eventually incorporated into the model as a way to improve the predictive performance of the model. AUC refers to the area under the ROC (receiver operating characteristic) curve which is usually utilized for testing the accuracy of a model, and it is not affected by the proportion of subjects in the analyzed sample (Parodi et al., 2022). The accuracy of the model is assessed based on the AUC value, the magnitude of which is proportional to the predictive performance of the model. The model accuracy of AUC value prediction can be categorized into four levels: excellent (0.9-1), good (0.8-0.9), fair (0.7-0.8), and poor (<0.7) (Swets, 1988).

### 2.4 Classification of suitable areas

The maximum test sensitivity plus specificity threshold (MTSPS) is usually used as a dividing line to delineate the suitable area of a species (Wang et al., 2024). The range is from 0 to MTSPS values for unsuitable areas and from MTSPS values to 1.0 for suitable areas. The MTSPS value of this study was 0.362, so areas with P ≤ 0.362 were classified as suitable areas for Astragali Radix, and areas with P>0.362 were classified as unsuitable areas. The predictions were imported into ArcGIS 10.7 software and the reclassification tool was used to categorize the potentially suitable areas, and the corresponding spatial areas were calculated and visualized.

### 2.5 Centroids of suitable areas

Mean centers of mass (centroids) for the suitable areas were calculated for each period using the Metrics Geographic Distribution tool in the Spatial Statistics tool of the ArcGIS 10.7 software. Combine centroids from different time periods into one vector data and plot migration directions and distances using the Point Set to Line tool in the Data Management tool.

## 3 Results

### 3.1 Model optimization results and accuracy evaluation

**Figure 2:**
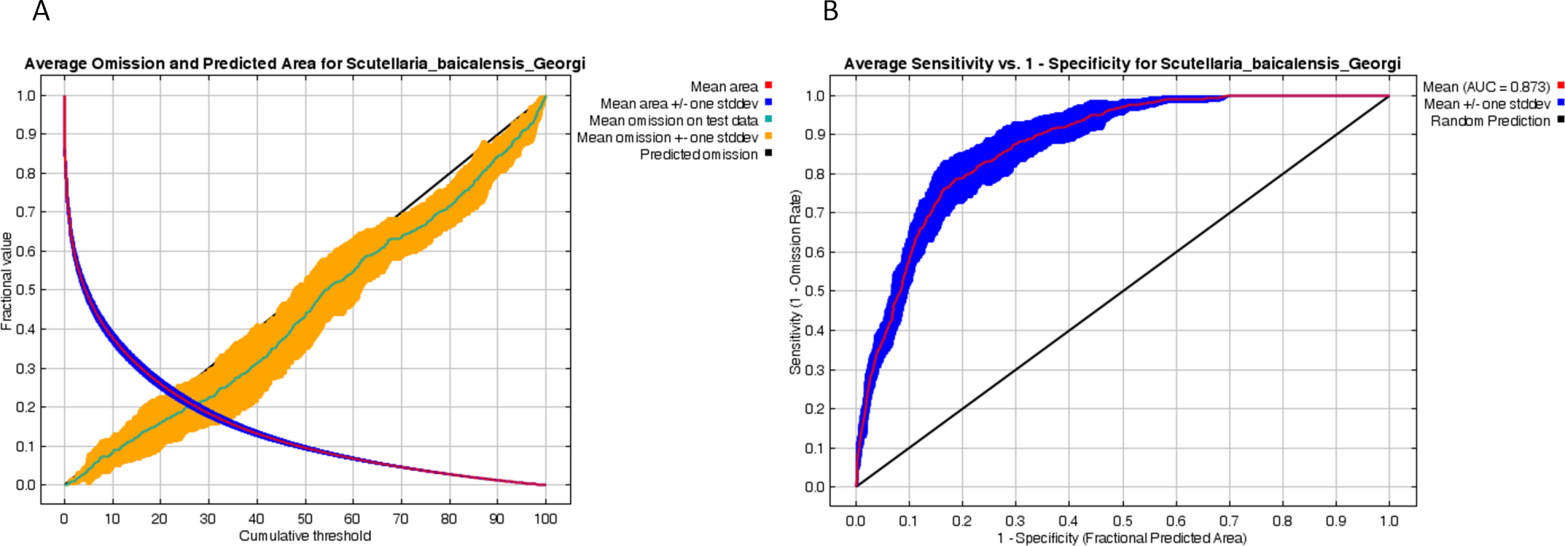
Model accuracy evaluation. AICc value of parameter combinations based on the ENMeval calculation. AICc, Akaike information criterion correction; L, linear; Q, quadratic; H, hinge; P, product; T, threshold. H, L, LQ, LQH, LQHP and LQHPT mean different feature categories. DAICc = 0 means the model with this parameter combination is the optimal one. ROC curves of MaxEnt result. **(A)** The ROC verification curve of Maxent model; **(B)** Jackknife test of the importance of variables.

When RM was 3.5 and FC was LQH, the DAICc was 0 and the AUC value was as high as 0.889, indicating that the model had been optimized for better prediction accuracy and less overfitting. The model has higher accuracy in predicting the suitable growth area of Astragali Radix under different climatic conditions (**Supplementary Figure S1**; **Figure 2**).

### 3.2 Influence of major environmental factors

**Figure 3:**
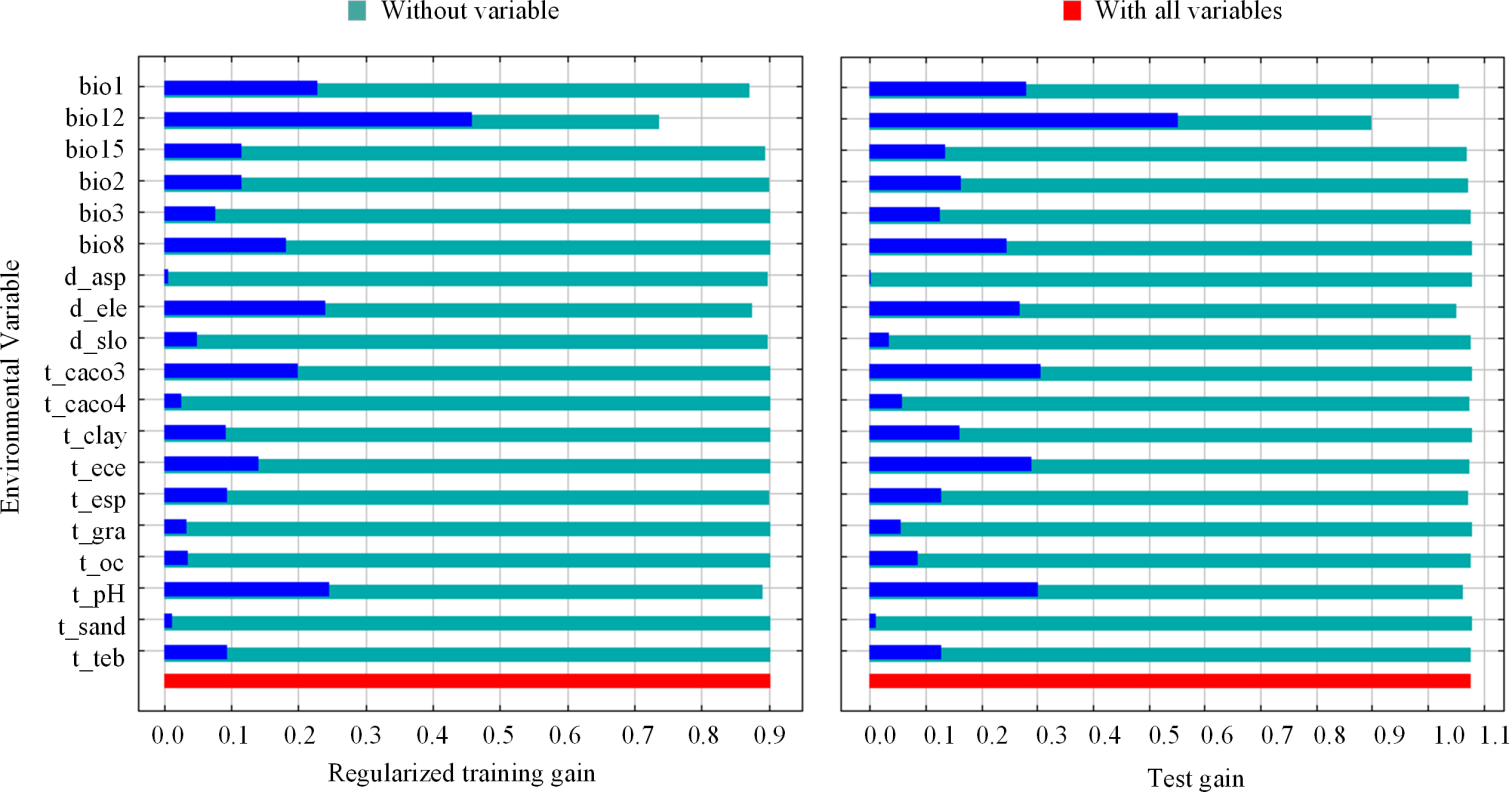
Results of jackknife test for the importance of the variables for MaxEnt.

Of the 19 environmental variables that were ultimately included in the model, those with a contribution greater than or equal to 5% were bio12 (annual precipitation, 41.6%), d_ele (elevation, 15.9%), t_caco3 (topsoil calcium carbonate, 14.8%), bio1 (annual mean temperature, 8.3%), bio15 (precipitation seasonality, 8%), t_ph (topsoil pH, 6%). In addition, the jackknife test showed that bio12, d_ele, t_caco3, bio1, bio15 and t_ph all fit the training data well, indicating that they contained the most useful information not contained in the other variables (**Figure 3**).

**Figure 4:**
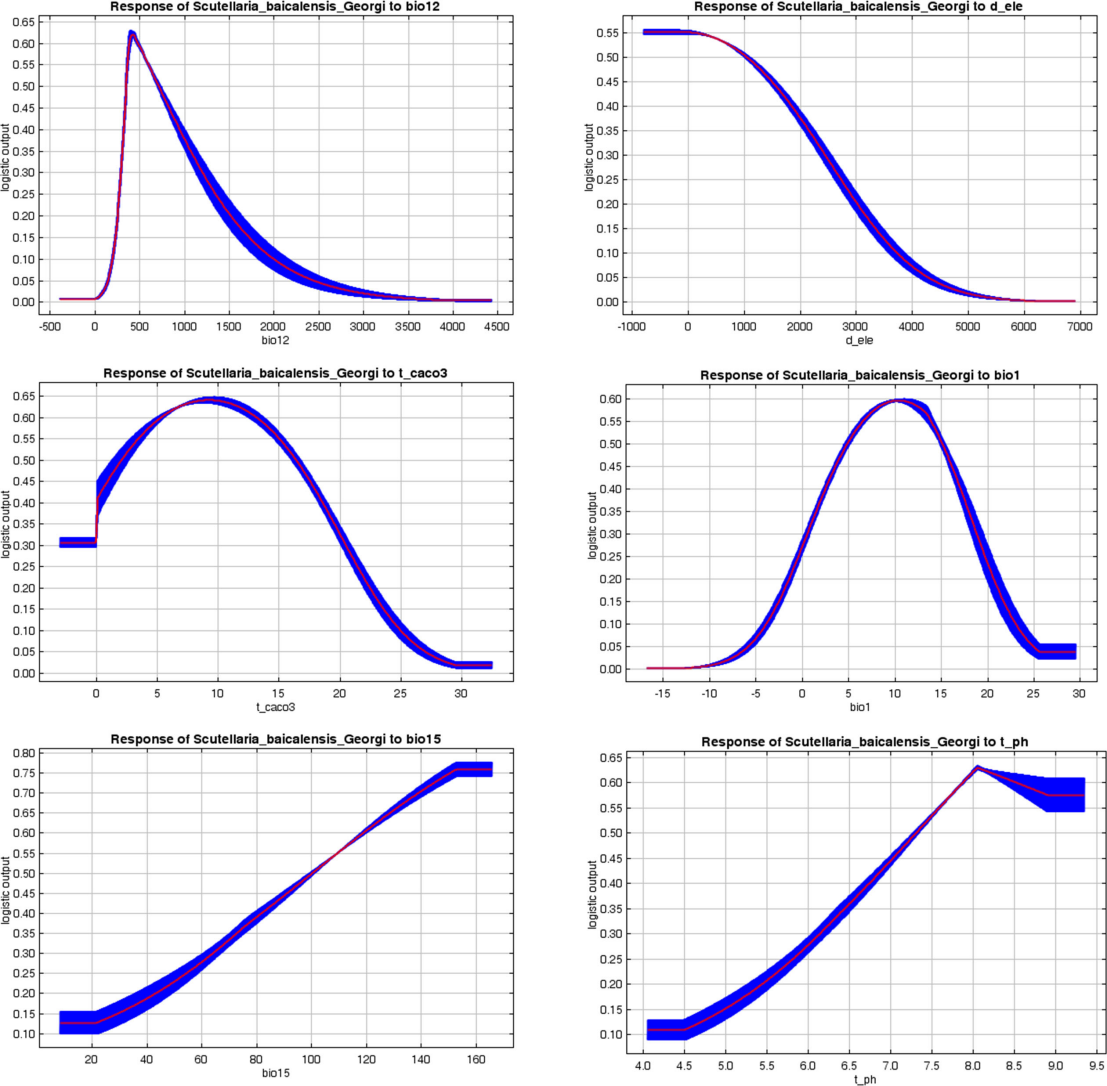
Response curve of Astragali Radix existence probability to the main factors.

The existence probability greater than and equal to 0.5 was taken as the most suitable condition for survival. From the one-factor response curve, it can be seen that when bio12 in the range of 351.96~712.77mm, d_ele in the range of -783.10~1052.71m, t_caco3 in the range of 2.08%~16.38%, bio1 was in the range of 4.86~15.08°C, bio15 in the range of -165.68~100.23, t_ph in the range of 7.31~9.34 was most suitable for growth of Astragali Radix (**Figure 4**).

### 3.3 Potential distribution of Astragali Radix in China

**Figure 5:**
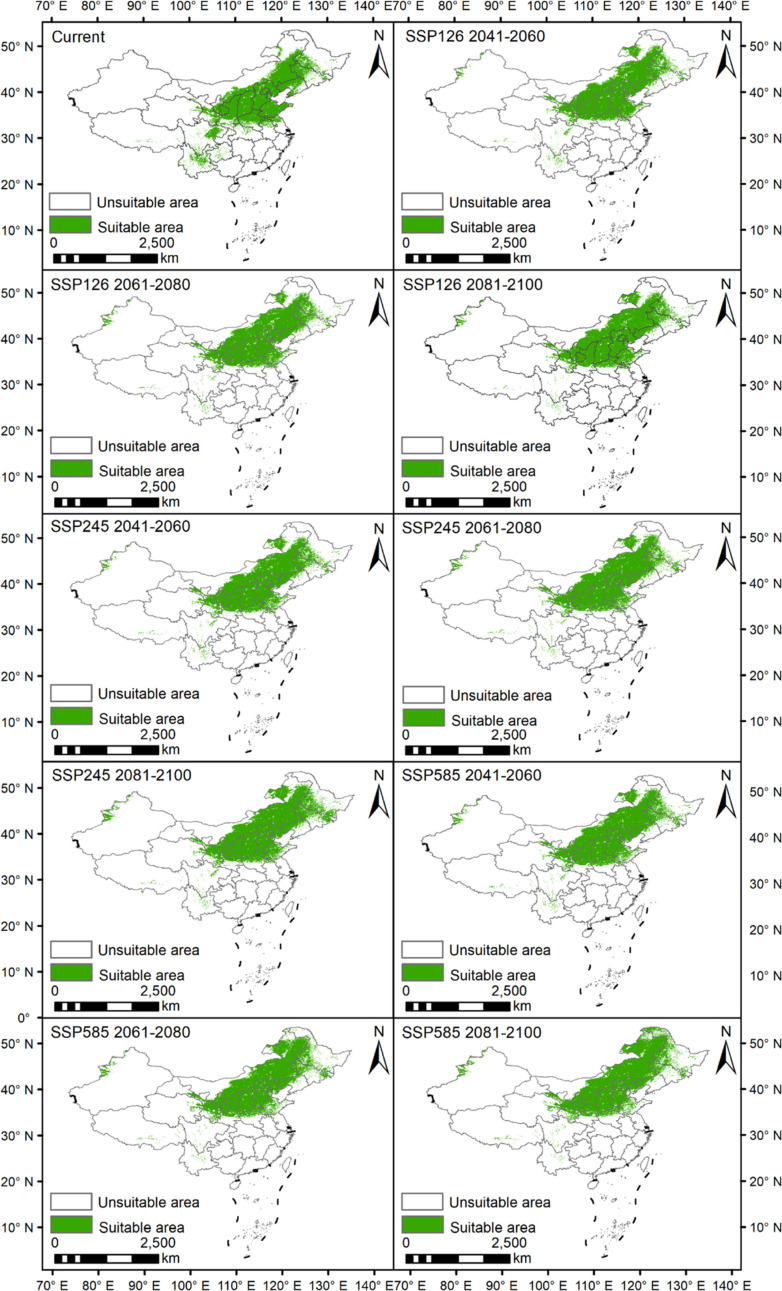
Prediction of potential suitable areas of Astragali Radix in different periods.

The results showed that under the current climatic condition, the suitable areas of Astragali Radix was 188.41×10^4^ km^2^, which was mainly concentrated in North China such as Beijing, Liaoning, Hebei, Shandong, Shanxi, Shaanxi, Henan, and Southwest China, such as Yunnan, Guizhou, and Sichuan. Under the SSP126 scenario, the suitable areas increased from 187.58×10^4^ km^2^ to 190.96×10^4^ km^2^ and finally reached 193.70×10^4^ km^2^. Under the SSP245 scenario, the suitable areas increased from 191.59×10^4^ km^2^ to 195.09×10^4^ km^2^ and finally reached 213.02×10^4^ km^2^. Under the SSP585 scenario, the suitable areas increased from 198.46×10^4^ km^2^ to 203.74×10^4^ km^2^ and finally reached 212.70×10^4^ km^2^ (**Supplementary Table S1**). The area of potential suitable areas is higher than that under the current climate conditions under all three climate scenarios. Under the same climate scenario, the area of suitable areas showed an increasing trend over time. In addition, the potential suitable areas for Astragali Radix under the three climate scenarios in the future will still be mainly distributed in North China, the suitable areas in Southwest China will be less than the current area, and new suitable areas will be developed in Northwest Xinjiang (**Figure 5**).

### 3.4 Dynamics of the future area of suitable areas

**Figure 6:**
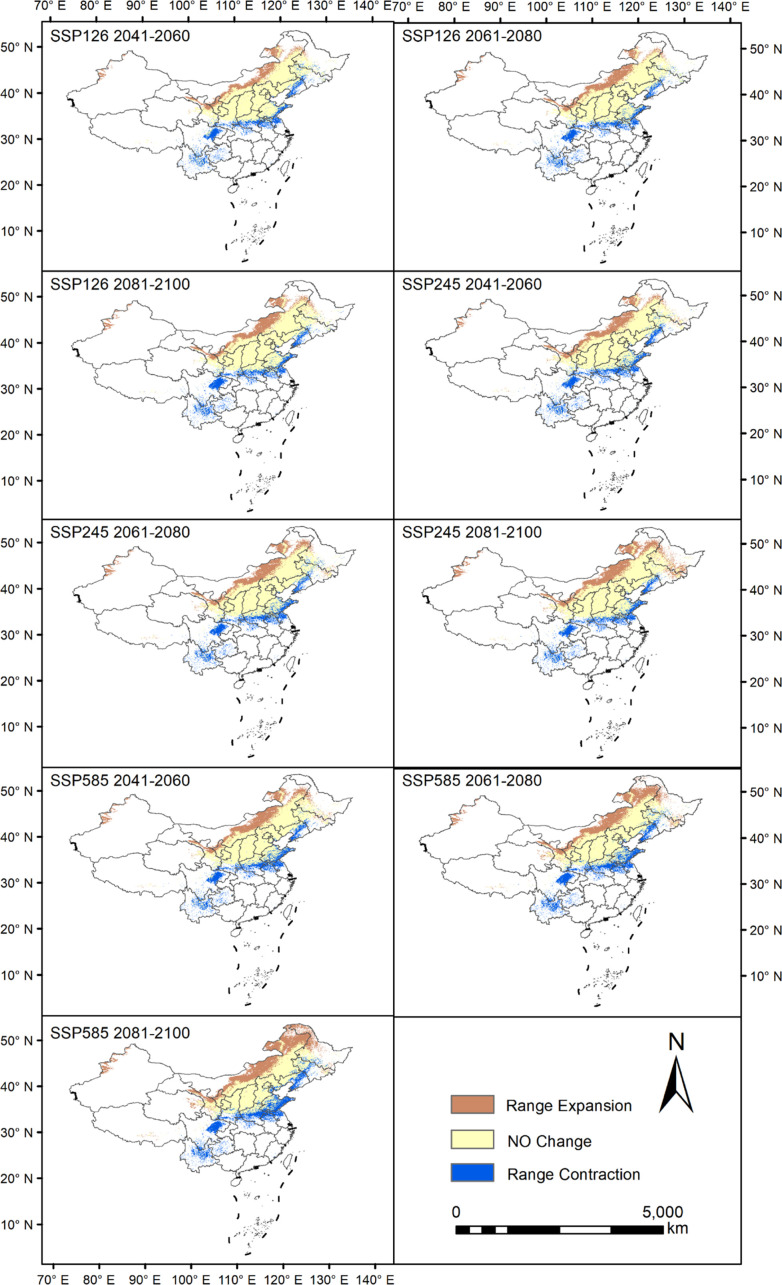
Suitable distribution changes of Astragali Radix in different periods.

The results showed that under the SSP126 scenario, the suitable areas showed a slight contraction (-0.84×10^4^ km^2^) during the period 2041-2060, followed by an expansion of 2.55×10^4^ km^2^ and 5.29×10^4^ km^2^ during the periods 2061-2080 and 2081-2100, respectively. Under the SSP245 scenario, the suitable areas will expand by 3.19×10^4^ km^2^ during 2041-2060, 6.68×10^4^ km^2^ during 2061-2080, and significantly by 24.61×10^4^ km^2^ during 2081-2100. Under the SSP585 scenario, the suitable areas will expand by 10.05×10^4^ km^2^ during 2041-2060, 15.33×10^4^ km^2^ during 2061-2080 and 24.30×10^4^ km^2^ during 2081-2100 (**Supplementary Table S2**). The expansion area is mainly located in Inner Mongolia Autonomous Region and northwestern Xinjiang, while the reduction area is mainly concentrated in the southwestern China. The suitable area of Astragali Radix has been expanding for most of the time in each period of the three climate scenarios compared with the current situation (**Figure 6**).

### 3.5 Centroids migration of suitable areas in the coming period

**Figure 7:**
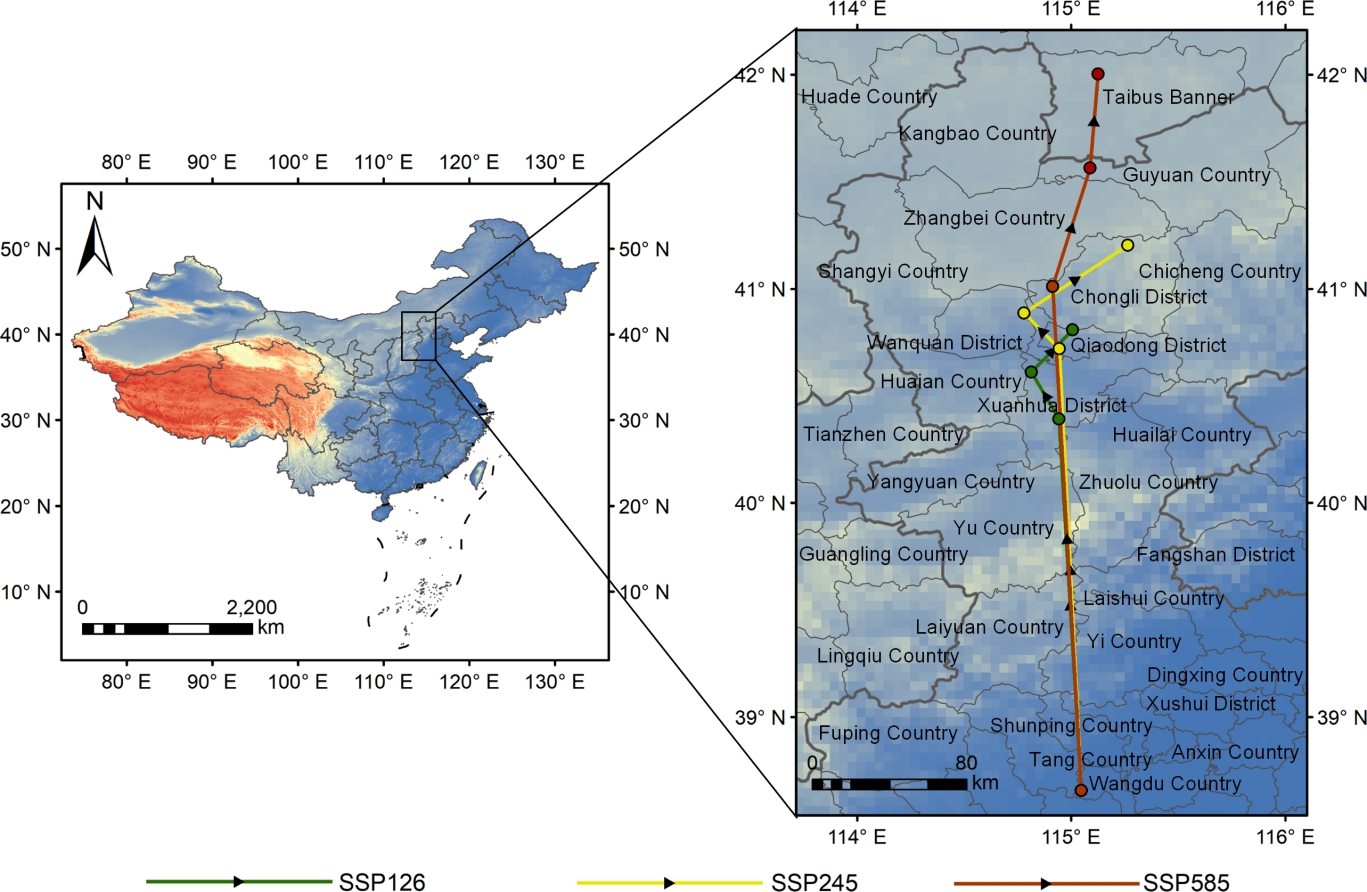
Situation of centroid shift of suitable areas of Astragali Radix in different periods.

Currently, the centroid of the suitable area of Astragali Radix is located in Tang County, Hebei Province (115.047°E, 38.656°N). From now to 2041-2060, the centroids under the three scenarios of SSP126, SSP245, and SSP585 has moved by 193.02 km, 229.55 km, and 261.88 km, respectively, and the centroid is located in Xuanhua District (114.943°E, 40.391°N), Qiaodong District (114.944°E, 40.720°N), and Chongli District (114.914°E, 41.011°N). From 2041-2060 to 2061-2080, the centroid shifted by 26.71 km, 23.11 km, and 63.20 km, and the centroids were located in Xuanhua District (114.814°E, 40.611°N), Qiaodong District (114.779°E, 40.887°N), and Chongli District (115.088°E, 41.564°N), respectively. From 2061-2080 to 2081-2100, the centroid shifted by 27.25 km, 53.81 km, and 48.92 km, and the centroids were located in Qiaodong District (115.006°E, 40.808°N), Chongli District (115.265°E, 41.204°N), and Taibesiqi (115.125°E, 42.004°N), respectively. Under all three climate scenarios, the suitable areas of Astragali Radix showed a northward trend, but the degree of northward movement was different, as shown by SSP585 > SSP245 > SSP126 (**Figure 7**).

## 4 Discussion

Astragali Radix is widely cultivated in China and its distribution is highly dependent on regional environmental conditions. In view of the continuing effects of climate change, it is necessary to assess the environmental influences on the distribution of Astragali Radix and to predict suitable distribution areas for the future. This study analyzed the changes in suitable areas of Astragali Radix based on the optimized MaxEnt model for the years 2041-2060, 2061-2080 and 2081-2100. More importantly, in order to avoid the overfitting problem of MaxEnt caused by the concentrated distribution points and cross-correlation among environmental variables, this study used the spatial analysis functions of ArcGIS and ENMTools to screen the distribution points and environmental variables. In addition, the key parameters RM and FC were optimized using the ENMeval software package in order to improve the prediction accuracy of the model. The AUC value of the optimized model was close to 0.9, indicating the reliability of the model for simulation and prediction. Therefore, the model can be used to predict the distribution of Astragali Radix.

Medicinal plants are affected by a variety of environmental factors during their growth and development. Wang et al. in their study of medicinal plants on the Tibetan Plateau noted that medicinal plants are greatly affected by temperature and precipitation (Wang et al., 2021). Astragali Radix, as a typical dry perennial herb, is suitable for growth in cool and dry climatic conditions. The drought environment inhibits the growth of aboveground parts of Astragali Radix, so that nutrients are preferentially transported to the roots, which affects the accumulation of flavonoid components, thus affecting the quality of Astragali Radi (Li, 2017; Liang et al., 2016). Elevation is also a major influence on the distribution of Astragali Radi in this study. Previous studies have suggested that when Astragali Radix is planted at an altitude of about 1700 m, it is more favorable for the accumulation of its main active components (Wu et al., 2021), and thus there will be a difference in the quality of Astragali Radix planted at different altitudes. Soil moisture is another important factor that affects the distribution of Astragali Radix, and it can have an impact on the activity and reproduction of Astragali Radix pests. For example, Lerin et al. found that high moisture environments promote the hatching of weevil eggs (Lerin, 2004). Excessive soil moisture can limit the growth length of the main root of Astragali Radix, reduce the formation of lateral roots, and also highly susceptible to root skin rot (Su, 2017). In addition to climatic and topographic factors, this study also found that soil calcium carbonate also affects the suitable area of Astragali Radix. It has been previously demonstrated that calcium carbonate not only restores the original pH of the soil, but also stabilizes soil pH through its acid-base buffering capacity (Yu et al., 2017). Therefore, this favors the growth of Astragali Radi, which prefers alkaline growth conditions (Wu et al., 2015). Moreover, previous studies have found strong anthropogenic correlations with medicinal plant diversity (Zhao et al., 2022a), suggesting that anthropogenic activities may also influence the regional distribution of Astragali Radix.

Intergovernmental Panel on Climate Change (IPCC) (2023) shows that compared to 1995-2014, the likely range of projected changes in global mean annual land precipitation over the period 2081-2100 is 0.0-6.6%, and the global mean surface air temperature average is likely to increase by 0.5-1.5°C (Intergovernmental Panel on Climate Change (IPCC) (2023)). Many studies have confirmed that climate change drives species migration to higher latitudes (Wang et al., 2023b; Bertrand et al., 2011). This is consistent with the overall northward migration of centroids in the Astragali Radix suitable areas. The specific migration direction and the variability of migration distance may be related to a variety of factors, such as human activities, altitude and land use type (Ding et al., 2020; Yang et al., 2013). Moreover, while previous studies have suggested that the frequency and intensity of extreme precipitation events may increase globally (Zhou et al., 2023) and that the geographic distribution of most plants may decrease (Wang and Wu, 2024). However, the area of suitable areas for Astragali Radix will expand in the future. This may be related to the fact that the main habitat of Astragali Radix is located in the Yellow River Basin area in northern China. Because some scholars have predicted that the precipitation in the Yellow River Basin will tend to decrease after the middle of the 21st century, which will undoubtedly favor the growth of Astragali Radix, which prefers to be dry (Liu et al., 2024).

In the future, the area of suitable area for Astragali Radix in Xinjiang shows an expanding trend, and the expanding area is concentrated in the northwestern oasis belt, which may be related to the change of oasis area (Jiao et al., 2024). Under global warming, the rate of alpine ice melt will accelerate in the future, potentially leading to an increase in oasis area. Meanwhile, in high-altitude and high-latitude environments, climate change is rapidly reducing the winter snowpack and accelerating the rate of spring snowmelt (Fyfe et al., 2017; Musselman et al., 2021). Therefore, the dynamics of oases in the context of climate change need to be fully taken into account when planning Astragali Radix cultivation in order to realize the long-term benign development of the cultivation. In addition, the provinces of Yunnan, Guizhou and Sichuan are located in southwest China, which have formed a unique “microclimate zone” due to their similar geographic location and natural conditions, and have been one of the major production areas of Chinese herbal medicines since ancient times (Liu, 2018). However, this study found that the area of suitable area for Astragali Radix in Southwest China showed a decreasing trend. Previously, some scholars predicted a significant increase in temperature and precipitation in Southwest China in the future (Jin et al., 2022; Liu et al., 2022), as well as the frequent occurrence of extreme weather and events in the region due to complex topographic conditions. Together, these factors constitute a potential threat to the growth environment of Astragali Radix, and thus Astragali Radix may need to migrate to areas with higher latitudes and relatively lower temperatures and humidity to adapt to the new climatic environment. This study predicted the current and future potentially suitable areas of Astragali Radix. The results can provide some basis for the screening of ecological planting areas and the control of key ecological factors for Astragali Radix in China. Nevertheless, the suitable areas of plant species depends not only on ecological conditions, but also on other factors such as economic development, government policies, and anthropogenic disturbances, and more types of environmental data need to be collected to enhance the applicability of the model predictions in future studies.

## 5 Conclusion

Our study identified precipitation, elevation, temperature, topsoil calcium carbonate and topsoil pH are the key environmental variables regulating the growth of Astragali Radix. Currently, the suitable area of Astragali Radix in China is mainly concentrated in North China and Southwest China, but the area of suitable area in Southwest China will be reduced in the future, and Northwest Xinjiang may develop into a new suitable area. In addition, the centroid position of the suitable area of Astragali Radix shows a northward trend, indicating that the population of Astragali Radix may migrate to higher latitudes to cope with the environmental changes caused by global warming. This study provides a scientific basis for the development of planting strategies and spatial distribution management of Astragali Radix, and helps to optimize the selection of areas for herbal medicine cultivation and resource conservation.

## Data Availability

The original contributions presented in the study are included in the article/**Supplementary Material**. Further inquiries can be directed to the corresponding authors.
