# Supplementary material for: Predicting the potential distribution of Astragali Radix in China under climate change adopting the MaxEnt model
Source: Front Plant Sci. 2024 Dec 6;15:1505985. doi: 10.3389/fpls.2024.1505985 (PMC11659014; doi:10.3389/fpls.2024.1505985)
Supplement: Supplementary file 1 [file DataSheet1.docx]

Supplementary Material


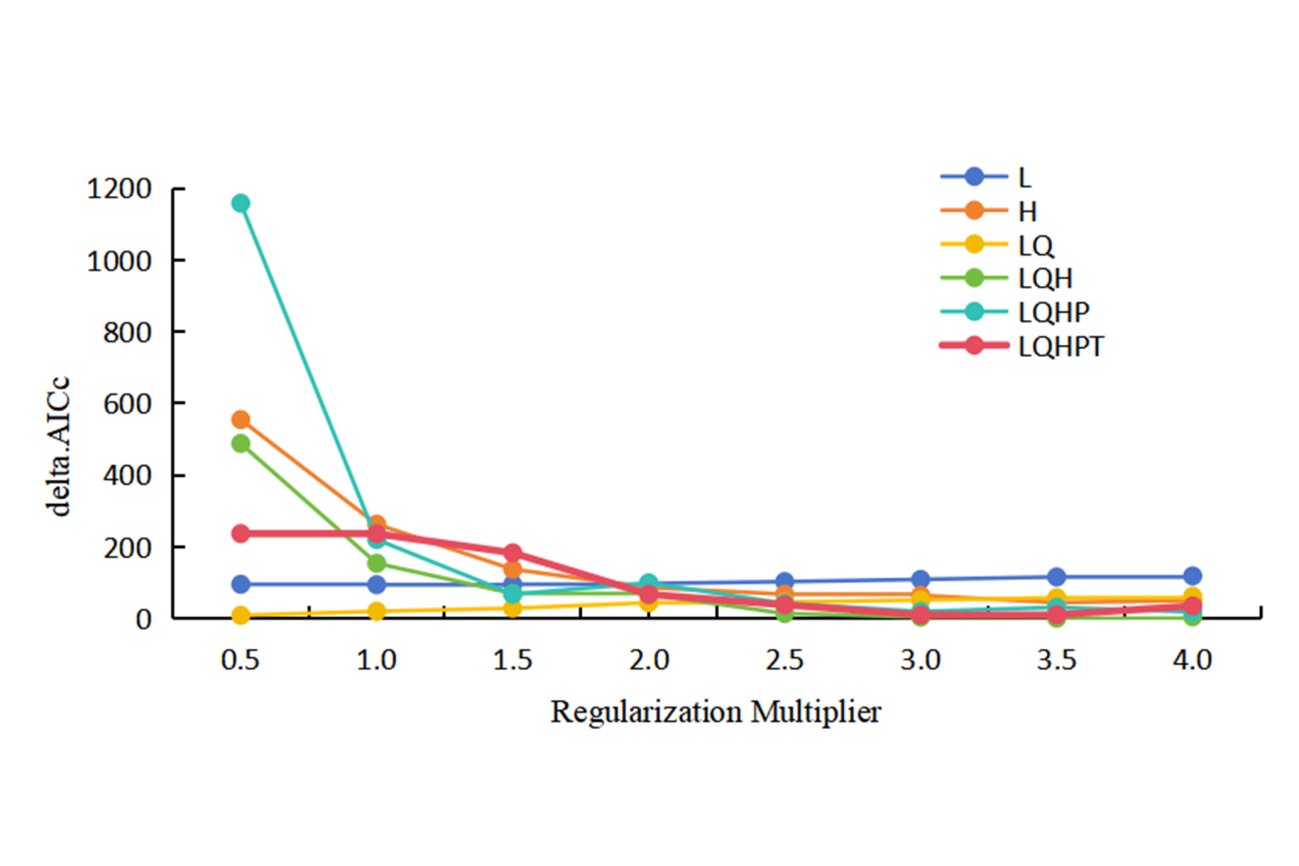


**FIGURE S1**  Model accuracy evaluation. AICc value of parameter combinations based on the ENMeval calculation. AICc, Akaike information criterion correction; L, linear; Q, quadratic; H, hinge; P, product; T, threshold. H, L, LQ, LQH, LQHP and LQHPT mean different feature categories. DAICc = 0 means the model with this parameter combination is the optimal one.

| Period | Area of suitable areas (10^4^ km^2^) | Area of unsuitable areas (10^4^ km^2^) |
| --- | --- | --- |
| Current | 188.41 | 771.59 |
| SSP126 2041-2060 | 187.57 | 772.43 |
| SSP126 2061-2080 | 190.96 | 769.04 |
| SSP126 2081-2100 | 193.70 | 766.30 |
| SSP245 2041-2060 | 191.59 | 768.41 |
| SSP245 2061-2080 | 195.09 | 764.91 |
| SSP245 2081-2100 | 213.02 | 746.98 |
| SSP585 2041-2060 | 198.46 | 761.54 |
| SSP585 2061-2080 | 203.74 | 756.26 |
| SSP585 2081-2100 | 212.70 | 747.30 |

**TABLE S1**  The area of suitable areas of Astragali Radix in different periods.

**TABLE S2**  Suitable distrtbution of Astragali Radix in different periods (10^4^ km^2^).

| Scenarios | Period | Aggregate change | Expansions | Unchanged | Contractions |
| --- | --- | --- | --- | --- | --- |
| SSP126 | 2041-2060 | -0.84 | 33.70 | 153.87 | 34.54 |
|  | 2061-2080 | 2.55 | 39.04 | 151.92 | 36.49 |
|  | 2081-2100 | 5.29 | 41.99 | 151.71 | 36.70 |
| SSP245 | 2041-2060 | 3.19 | 41.43 | 150.17 | 38.24 |
|  | 2061-2080 | 6.68 | 46.09 | 148.00 | 39.41 |
|  | 2081-2100 | 24.61 | 59.80 | 153.22 | 35.19 |
| SSP585 | 2041-2060 | 10.05 | 49.34 | 149.12 | 39.29 |
|  | 2061-2080 | 15.33 | 62.44 | 141.30 | 47.11 |
|  | 2081-2100 | 24.30 | 76.95 | 135.76 | 52.65 |
